# Mistranslation protects against lifespan reduction due to mating in *Drosophila melanogaster* females

**DOI:** 10.1093/g3journal/jkag216

**Published:** 2026-08-12

**Authors:** Luana Branco, William Yeung, Joshua R Isaacson, Amanda J Moehring

**Affiliations:** Western University Department of Biology, Western University, London, Ontario N6A 5B7, Canada; Western University Department of Biology, Western University, London, Ontario N6A 5B7, Canada; Western University Department of Biology, Western University, London, Ontario N6A 5B7, Canada; Western University Department of Biology, Western University, London, Ontario N6A 5B7, Canada

**Keywords:** mistranslation, tRNA, *Drosophila melanogaster*, ageing, reproduction, fertility

## Abstract

Accurate translation of genes into proteins is critical to organism fitness, and errors in this process are usually detrimental and cause proteotoxic stress. Mistranslation occurs when the amino acid that is incorporated into the nascent polypeptide chain does not match what is dictated by the genetic code. Valine-to-serine (V→S) and threonine-to-serine (T→S) mistranslating models of the fruit fly *Drosophila melanogaster* have demonstrated a surprising, sex-specific increase in virgin female longevity. We predict that the added stressor of reproduction would eliminate this mistranslation-induced lifespan increase since females prioritize reproductive tissues over somatic tissues, and proteotoxic stress would therefore lead to higher protein damage and cell death in the soma of mated females. We measured the impact of reproduction on V→S and T→S mistranslating *D. melanogaster* by measuring longevity, egg laying, and fecundity. Counter to our prediction, both V→S and T→S mistranslation led to a sex-specific increase in mated female longevity compared with nonmistranslating controls. Additionally, the risk of death decreased for mated females with mistranslation, beyond the pure additive benefits of mistranslation alone. These effects could not be explained by reduced egg laying or fertilization rates in mistranslating females. Thus, we find that the added proteotoxic stress caused by mistranslation does not exacerbate the detrimental effects of reproduction and instead can ameliorate lifespan decreases due to female reproduction.

## Teaser

Accurate translation of genes into proteins is critical to organism fitness, and errors in this process are usually detrimental. Counter to expectation, Branco et al. found that errors in translation led to a sex-specific increase in longevity of both unmated and mated females, with the greatest benefit in mated females. These effects could not be explained by reduced egg laying or fertilization rates in mistranslating females. Thus, the authors find that the added proteotoxic stress caused by mistranslation does not exacerbate the detrimental effects of reproduction and instead can ameliorate lifespan decreases due to female reproduction.

## Introduction

Reproduction has high physiological costs and investing in reproduction can shorten lifespan (reviewed in Maklakov and Immler 2016). According to the Disposable Soma Theory, this lifespan reduction is due to the costly allocation of energy toward gamete production at the expense of somatic maintenance (Kirkwood 1977; Kirkwood 1997; Kirkwood and Holliday 1997; reviewed in Flatt 2011). In *Drosophila melanogaster*, oogenesis and embryogenesis are tightly regulated processes with high energetic and nutritional needs. High metabolic investment is required from females to produce oocytes containing the yolk proteins, lipids, and carbohydrates necessary to undergo future embryogenesis (Gutzeit et al. 1994; Buszczak et al. 2002; Sieber et al. 2016). To support high metabolic needs, a high protein diet is often favored by mated females compared with males and unmated females (Zheng et al. 2025). In addition to high protein intake, female *D. melanogaster* invest energy into maintaining proteostasis in germline tissue at the expense of somatic tissue, prioritizing offspring investment over parental longevity (Fredriksson et al. 2012). The prioritization of germline protein quality control over somatic tissue in mated females should therefore lead to a more pronounced impact of proteotoxic stress on somatic traits, such as lifespan, compared with the same traits in males and unmated females.

Inaccurate translation of mRNA into proteins can produce misfolded proteins and disrupt proteostasis, compromising organismal health and fitness (Santos et al. 2018; Tavares et al. 2021). Mistranslation, defined as the erroneous incorporation of noncognate amino acids into a nascent polypeptide chain, has been primarily studied in vitro or using single-cell models (*Escherichia coli*, *Saccharomyces cerevisiae*, human cell lines: eg Samhita et al. 2020; Berg et al. 2021; Berg et al. 2022; Davey-Young et al. 2024; Zhang and Ling 2025). In these studies, mistranslation causes defects in protein production, increases in protein aggregation, proteotoxic stress, and growth defects (Paredes et al. 2012; McDonald et al. 2025; Tennakoon et al. 2025). Mistranslation is usually detrimental but can provide benefits in certain circumstances. In some yeast and bacterial species, alteration of the genetic code can confer an evolutionary advantage in evading host immune responses and developing antibiotic resistance (Pezo et al. 2004; Bacher et al. 2007; Silva et al. 2007; Vargas-Rodriguez et al. 2021; Schuntermann et al. 2023). Additionally, mistranslation has been shown to protect cells against oxidative stress, increase cell survival under UV-induced DNA damage, and upregulate the yeast longevity gene *PNC1* (Netzer et al. 2009; Silva et al. 2009; reviewed in de Pouplana et al. 2014; Fan et al. 2015; Samhita et al. 2020). Despite mistranslating transfer RNA (tRNA) variants being common in humans (Lant et al. 2018; Berg et al. 2019a; Hasan et al. 2023; Tennakoon et al. 2025) and mistranslation being implicated in disease (Bloom et al. 2006; Kalapis et al. 2015; Lant et al. 2018; Santos et al. 2018), our understanding of how it affects various tissues in multicellular organisms is limited by a scarcity of in vivo research using multicellular models.

Recent studies in *D. melanogaster* revealed that variant tRNA genes that induce valine-to-serine (V→S) or threonine-to-serine (T→S) mistranslation increased longevity in unmated female flies (Isaacson et al. 2024b). The underlying mechanism for this increase in longevity is unknown but may be due to mistranslation activating stress-response pathways, such as those affecting the cell cycle and DNA repair (Isaacson et al. 2024a), causing a general protective effect against other stressors that could impact longevity. However, reproduction would shift cell maintenance toward reproductive tissues at the expense of somatic tissues, and gamete production and maturation require increased protein synthesis activity and reallocation (Bownes et al. 1991; Ma et al. 2022). We therefore predict that mistranslation's beneficial effect on unmated female lifespan will be removed when females are reproducing, and that their lifespan will be shorter than controls due to the combined detrimental effects of prioritizing germline tissue maintenance at the expense of somatic tissue, proteotoxic stress from mistranslation, and energetic costs of reproduction (Salmon et al. 2001; Harvanek et al. 2017).

To test this prediction, we assess the effects of mating and mistranslation on *D. melanogaster* lifespan. We found that reproduction decreases longevity in females, as expected, but that tRNA-induced mistranslation buffers its negative impact, counter to prediction. Mistranslation does not affect egg laying or fertilization, and therefore the increase in longevity in mistranslating mated females is not due to failure of basic reproductive output. Hence, mistranslation increases lifespan even in the presence of the additional proteotoxic burdens placed on females because of mating.

## Methods

### Fly husbandry and stocks

All flies were housed at 24 °C, 75% relative humidity (RH), 14:10 light:dark (L:D) (longevity assay) and 25 °C, 80% RH, 12:12 L:D (egg laying and fecundity assays). Flies were housed on Bloomington standard cornmeal agar food media recipe (Bloomington Drosophila Stock Center; https://bdsc.indiana.edu/information/recipes/bloomfood.html). Mistranslating flies were created using a tRNA model first developed in *S. cerevisiae* (Berg et al. 2017, 2019b). In *D. melanogaster*, a tRNA^Ser^ containing the anticodons for valine (AAC) or threonine (AGU), and a G26A secondary mutation, were previously integrated into a set landing site on the second chromosome (Isaacson et al. 2024b). These tRNAs will recognize valine or threonine codons whilst remaining serylated, resulting in valine-to-serine (V→S) or threonine-to-serine (T→S) mistranslation. Mistranslation was confirmed via mass spectrometry (Isaacson et al. 2024b). Mistranslating stocks had FRT sites flanking the mistranslating tRNA sequence, allowing for the removal of the tRNA gene in the presence of flippase to create control flies (Gronostajski and Sadowski 1985; Isaacson et al. 2024b). The control flies have an identical genetic background to the mistranslating flies, with the only difference being the removal of the mistranslating tRNA. The genotype of the flies with the mistranslating tRNA is: *w^1118^; P{CaryP}-attP40^wmw+=pattB-tRNA^/CyO; MKRS/TM6B*. The genotype of the control flies is: *w1118; P{CaryP}-attP40^wmw+=pattB-FLP-out^/CyO; MKRS/TM6B.* Wildtype *Canton S* (*CS*) males were used as a control mate for the egg laying and fecundity assays.

### Longevity assays

Longevity assays were conducted as described in Isaacson et al. (2024b). We collected equal numbers of virgin males and females of each mistranslating line (V→S and T→S) and their controls under light CO_2_ anesthesia within 8 h of eclosion, removed any with visible signs of deformity (following the protocol in Isaacson et al. 2022), separated by sex, and placed in new food vials. For each mistranslating line, virgin flies were divided into replicates of 8 vials each, with each vial within the replicate containing 4 to 8 (V→S) or 4 to 6 (T→S) flies (Fig. 1; Supplementary Fig. 1). Four vials contained unmated flies, containing a single sex (male or female) and genotype (mistranslating or control). Four vials contained mated flies, containing equal numbers of males and females, with the total number being equal to that for the unmated flies, for each genotype (mistranslating or control). All vials set up on a given day contained the same number of flies per vial across all groups to minimize environmental effects (Austin et al. 2014). For example, if on a given day there were 4 unmated flies per vial, then there would be 2 corresponding vials that each contain 2 mated males and 2 females (Supplementary Fig. 1); if on another day, there were 6 unmated flies per vial, then there would be 2 mated vials that each contain 3 males and 3 females. Flies that escaped or were killed during transfer were censored from the observation. In total, 82 flies from each group were tested from the V→S cohort and 80 were tested from the T→S cohort (the breakdown of vials and flies is listed in Supplementary Table 1). Every 3 d, flies were transferred to new food and deaths were recorded.

**Fig. 1.**
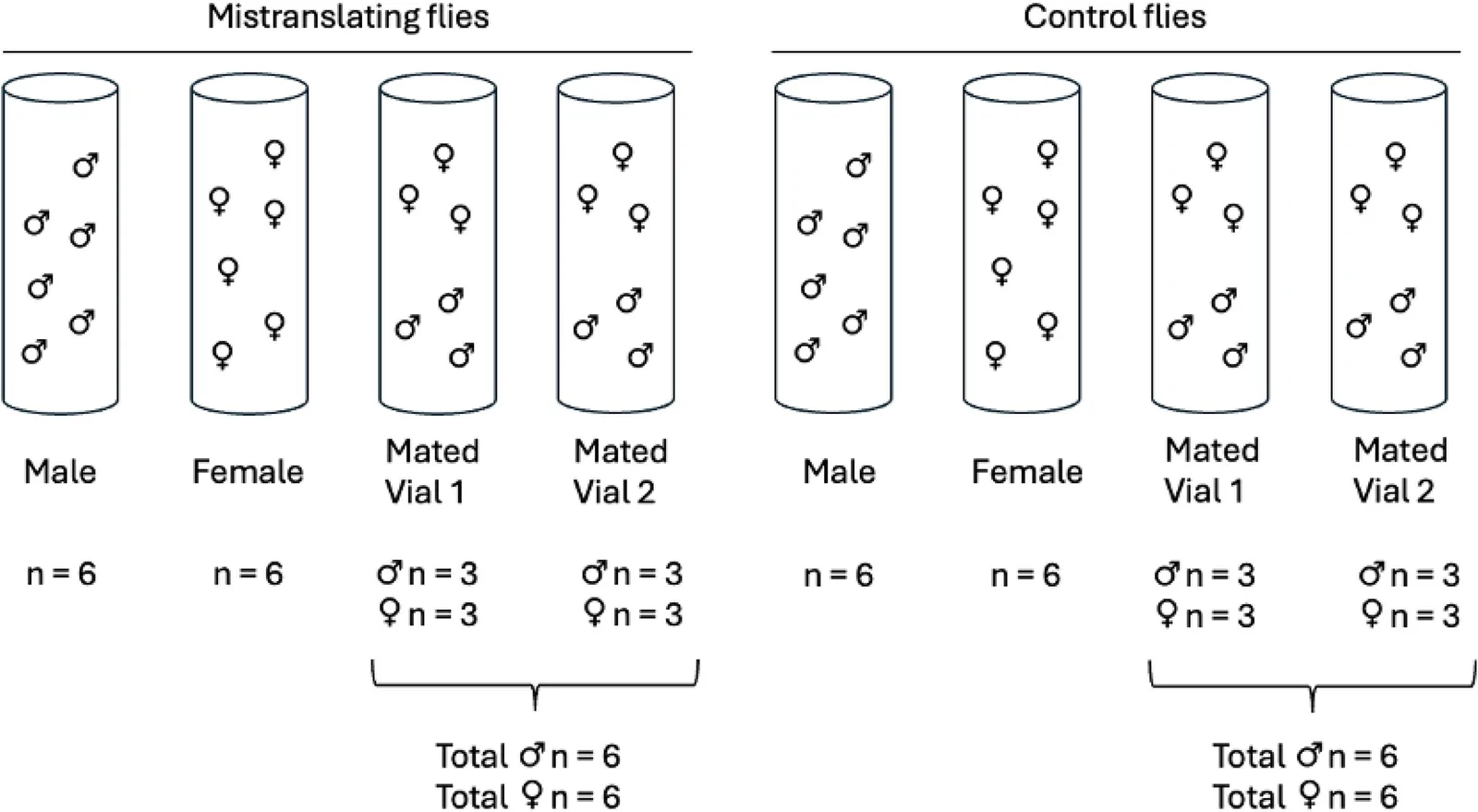
Longevity assay setup on a given day. Each male is depicted in the vials by the “♂” symbol and females are depicted by the “♀” symbol. The number of each fly in each vial is displayed beneath the vial. For a given set, vials contained a total of 4, 6, or 8 flies, with all vials within that set having the same number. The example of a total of 6 flies is shown; for examples of 4 and 8 flies see Supplementary Fig. 1.

### Egg laying and fertilization assays

Virgin wildtype *CS* males and virgin mistranslating and control females were collected under light CO_2_ anesthesia and separated by sex. Flies were aged 3 to 7 d prior to being placed in single mating pairs and observed for up to 2 h until mating occurred; unmated pairs were discarded. We transferred females 20 to 30 min postmating to a fresh 35 × 10 mm grape juice plate (Nouhaud et al. 2018) containing 10–30 mg of 1:1 ddH_2_O and yeast mixture added to the surface. We allowed females to lay eggs for approximately 24 h, transferred females to a new plate, and counted the number of eggs each female laid up to 3 h past lights on (Nouhaud et al. 2018). To assess eggs that were fertilized from sperm coming from short-term sperm storage vs long-term sperm storage, we assessed egg laying on days 1 to 4 and 7 to 10 postmating, respectively (Dhillon et al. 2020). Females were transferred to new grape juice plates every 24 h for the days that egg counting was performed; in between the short-term and long-term sperm storage observation, female flies were kept on Bloomington standard cornmeal agar food media. For V→S genotypes, we assessed egg laying for 8 control females and 10 mistranslating females; for T→S genotypes, we assessed 12 control females and 11 mistranslating females. To confirm fertilization of eggs in both short-term and long-term sperm storage periods, we examined the fertilization status of the eggs laid by mistranslating and control flies. We considered eggs to be fertilized if they showed visible cellularization when assessed under a stereo microscope (Lecuit and Wieschaus 2000). For V→S genotypes, we assessed 20 eggs chosen at random across all the females for their fertilization status. To randomize the egg selection, we numbered all eggs and used a random number generator to determine which 20 would be selected. For T→S females, we assessed all eggs from all females for their fertilization status.

### Statistical analyses

We performed the statistical analysis and graphing in RStudio version 2026.01.1 + 403 running R 4.5.2 unless otherwise noted. We created longevity Kaplan–Meier curves (*survival* and *survminer* packages in R) as described in Isaacson et al. (2024b) and adjusted using the Benjamini–Hochberg (BH) false discovery rate (FDR) procedure (Supplementary Table 2; Benjamini and Hochberg 1995). To analyze the interaction effects between mistranslation and mating, we used Cox proportional hazard models (*survival* package in R). We included initial vial population size as a covariate to control for crowding effects. Additionally, we tested for random effects across individual vials by including vial ID as a frailty term; however, vial ID had negligible effects in both the V→S and T→S models (frailty variance estimate = 5 × 10^−7^ and 5 × 10^−3^, LRT *P* = 0.88 and 0.27, respectively) and so was excluded from downstream analyses. When verifying proportional hazards assumptions, we found that the Schoenfeld residual plot of mistranslation exhibited time-dependency; thus, we included a time-transformation term for mistranslation as an additional covariate. We adjusted *P*-values for multiple testing of covariates across Cox models using the BH FDR procedure, as above for longevity. Lastly, we calculated the confidence intervals and standard deviations of survival medians in Microsoft Excel Version 16.101.1.

Egg laying analysis was performed using linear mixed effects (*nlme* package in R) to assess the effects of mistranslation, sperm storage period, and their interaction on egg laying behavior. We used a square root transformation to normalize the egg laying data and included fly ID as a random intercept to control for random effects from within-fly variability. We then obtained the estimated marginal means and their standard errors (*emmeans* package in R) for visualization and for calculation of % differences between groups. We used a Wilcoxon test to analyze long-term and short-term egg laying when either pooled or separated by sperm storage periods. All data figures were generated in R using *ggplot2*.

## Results

We assayed *D. melanogaster* that express either a tRNA that mistranslates valine-to-serine (V→S) or threonine-to-serine (T→S) (Isaacson et al. 2024b) to determine the effects of mistranslation on lifespan when flies are unmated vs mated (Fig. 1; Supplementary Fig. 1 and Supplementary Table 1). Control flies were created by using flippase to hop out the V→S and T→S mistranslating tRNA, controlling for any background genetic effects that may have arisen in these fly strains over time. As a result, the controls are genetically identical to the assay groups save for the absence or presence of the mistranslating tRNA. We first compared the longevity of mated and unmated flies to confirm the known effect of mating on reducing female lifespan (Kirkwood and Holliday 1997; Fredriksson et al. 2012; Koliada et al. 2020). As expected, female flies containing a V→S or T→S mistranslating tRNA and their associated controls had significantly decreased longevity due to mating (*P* < 0.0001 for all comparisons; Table 1 and Fig. 2; Supplementary Table 2). In contrast, males with a V→S or T→S mistranslating tRNA and their associated controls did not have a significant decrease in lifespan due to mating (Table 1 and Fig. 2; Supplementary Table 2). This aligns with the observation of Koliada et al. (2020) that reproduction causes a stronger detrimental effect in female compared with male lifespan.

**Fig. 2.**
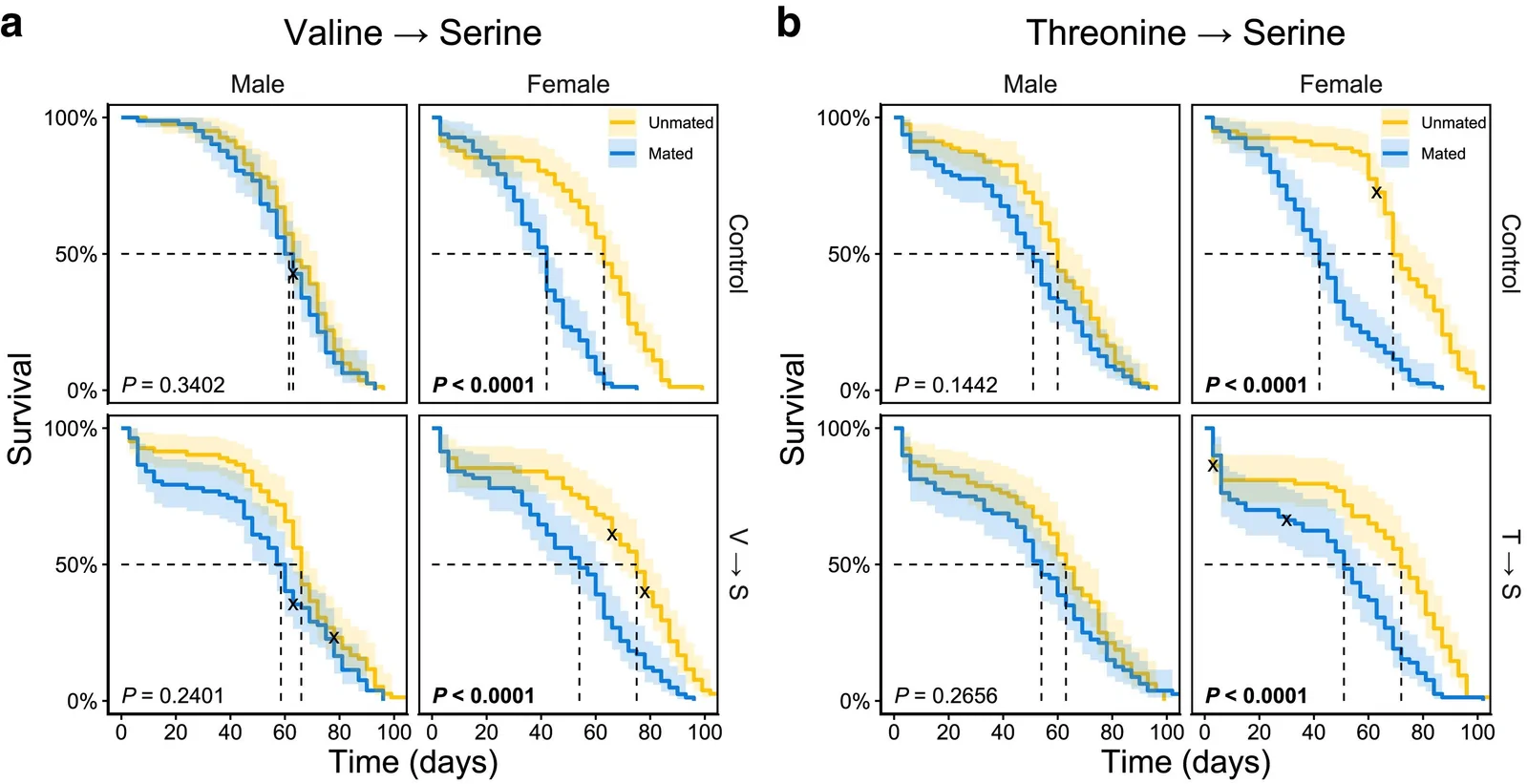
Survival of unmated (blue) vs mated (yellow) male or female flies for: a) V→S and b) T→S mistranslating males and females and corresponding controls. Shaded regions represent the 95% confidence intervals and × symbols represent flies that were censored from the observation (ie loss during transfer). 50% survival is displayed by dashed lines. Significant *P*-values are shown in bold. Note that these are the same data as in Figs. 3 and 4, with different grouping. Survival was compared using Fleming-Harrington tests corrected using the BH procedure.

**Table 1.** Median lifespan for each genotype tested according to mistranslation model, sex, and mating status.

Model	Sex	Mating status	Genotype	Median lifespan (days)	SD	95% CI
V→S	F	Unmated	Mistranslating	75	30.053	6.545
			Control	63	25.018	5.448
		Mated	Mistranslating	54	26.917	5.862
			Control	42	16.718	3.641
	M	Unmated	Mistranslating	66	23.805	5.184
			Control	63	16.666	3.629
		Mated	Mistranslating	57	27.764	6.046
			Control	60	17.336	3.775
T→S	F	Unmated	Mistranslating	72	32.259	7.025
			Control	69	23.118	5.034
		Mated	Mistranslating	51	27.803	6.055
			Control	42	19.904	4.335
	M	Unmated	Mistranslating	63	28.390	6.183
			Control	60	23.059	5.022
		Mated	Mistranslating	54	29.709	6.470
			Control	51	24.990	5.442

We next assessed whether the lifespan improvement previously seen for mistranslating unmated females (Isaacson et al. 2024b) is eliminated when females divert investment into reproduction, if any observed effects are also present in males, and if effects are present for both types of mistranslation. To this end, we compared the survivorship of mistranslating lines and their controls for each sex for V→S and T→S mistranslation (Table 1 and Fig. 3; Supplementary Table 2). V→S mistranslation increased the longevity of both unmated (*P* < 0.0001) and mated (*P* < 0.0001) females compared with their controls (Fig. 3a). These results indicate that mistranslation from V→S leads to an increase in the longevity of female *D. melanogaster*, regardless of mating status. One consideration is whether the results are simply due to survivor bias: if the “weakest” mistranslating flies are those that do not survive to adulthood, then the adults scored for longevity may be a skewed sample that causes the average lifespan to be greater than in wildtype flies. However, survivor bias would only affect the average lifespan and would not affect the maximum of the distribution; in other words, it would not cause the appearance of flies whose longevity is outside of the distribution observed for wildtype flies. We can therefore rule out survivor bias as the cause of the increased longevity since the longest-lived flies (the furthest tail of the distribution) are all mistranslating flies. In contrast, mistranslation from V→S did not have a significant impact on unmated and mated male longevity (Fig. 3a), further demonstrating that V→S mistranslation has sex-specific effects. Unlike what was seen in V→S, mistranslation from T→S in unmated females and males did not have a significant effect on longevity when compared with their respective controls (Fig. 3b). However, similar to what was seen for V→S, mistranslation from T→S in mated females resulted in a significant increase in longevity when compared with control females (*P* = 0.0011), while males had no change in lifespan (Fig. 3b). This indicates that T→S mistranslation, like V→S, causes sex-specific increased longevity in mated females.

**Fig. 3.**
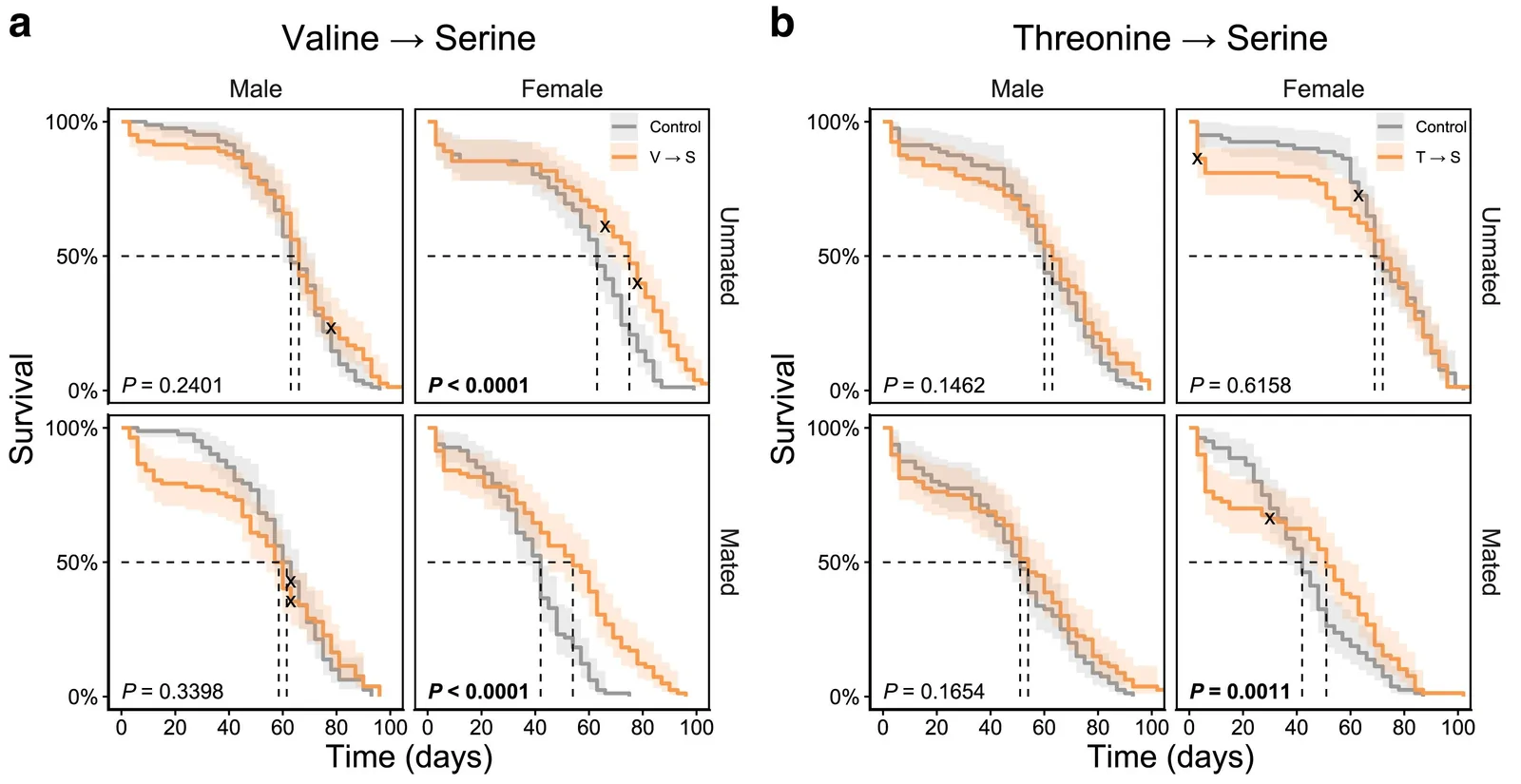
Survival of mistranslating flies (orange) vs their respective controls (gray) for male or female a) V→S or b) T→S mistranslation while mated or unmated. Shaded regions represent the 95% confidence intervals and × symbols represent flies that were censored from the observation (ie loss during transfer). 50% survival is displayed by dashed black lines. Significant *P*-values are shown in bold. Note that these are the same data as in Figs. 2 and 4, with different grouping. Survival was compared using Fleming-Harrington tests corrected using the BH procedure.

We next wanted to ask if the presence of mistranslation “rescues” mated female longevity to be the same length as for mated males; in other words, if it is sufficient to remove the sex-specific impact of mating on longevity. To determine the baseline effect without mistranslation in our flies, we compared the longevity of controls. Unmated control females and males did not differ in lifespan, while mated V→S and T→S control female lifespan was significantly shorter than mated control male lifespan (*P* < 0.001, *P* = 0.0049, respectively; Table 1 and Fig. 4; Supplementary Table 2). In other words, mating reduces lifespan in control females compared with males, as expected. When females are unmated, mistranslation significantly increases female lifespan to be greater than males in the presence of both V→S (*P* = 0.0079; Fig. 4a) and T→S (*P* = 0.014; Fig. 4b) mistranslation. When females are mated, mistranslation does not increase lifespan to be greater than males, but does “rescue” it to be the same as males, as we did not see a significant difference between mated female and male lifespan for either V→S (Fig. 4a) or T→S (Fig. 4b) mistranslation. Therefore, while mistranslation did not extend the lifespan of mated females beyond that of mated males, mistranslation prevented mated female lifespan from being significantly shorter than mated male lifespan, as is seen with their control counterparts. In other words, mistranslation buffers against the typical lifespan reduction seen in mated females (Burger and Promislow 2004). These results indicate that the presence of mistranslation provides an advantage to female longevity, regardless of mating status.

**Fig. 4.**
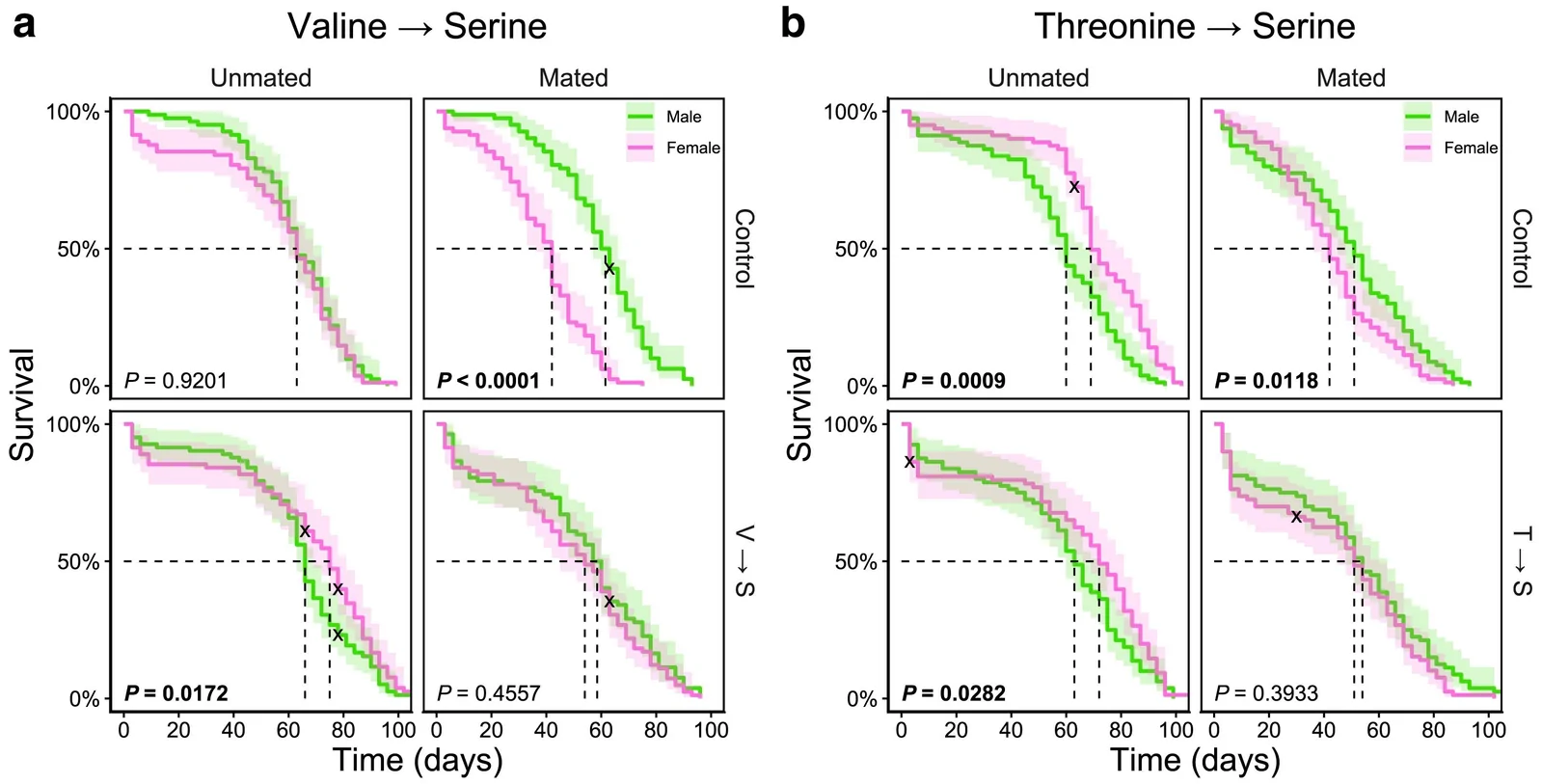
Survival of females (pink) vs males (green) for (a) V→S and (b) T→S mistranslating flies and controls while mated or unmated. Shaded regions represent the 95% confidence intervals and × symbols represent flies that were censored from the observation (ie loss during transfer). 50% survival is displayed by dashed lines. Significant *P*-values are shown in bold. Note that these are the same data as in Figs. 2 and 3, with different grouping. Survival was compared using Fleming-Harrington tests corrected using the BH procedure.

It is possible that the “buffering” effect is simply an additive increase in longevity that is present regardless of mating status. To assess if the effects of mating and mistranslation on longevity are additive vs synergistic, we analyzed predicted risk of death using Cox proportional hazard models. In alignment with the outcomes of our survival curve analyses, we found that mating increased risk in both V→S females (*P* < 0.0032; Supplementary Table 4) and T→S females (*P* < 0.0001; Supplementary Table 5). Our analysis also showed that the time-independent mistranslation covariate increases risk in both V→S females and T→S females (*P* < 0.0001 for both; Table 2), but that the time-dependent mistranslation covariate decreases risk in both (*P* < 0.0001 for both; Table 2). We calculated the time point at which the time-dependent and time-independent mistranslation covariates are equal (ie risk increase and risk decrease balance out for a net-zero risk) and predict that mistranslation, as a whole, begins to reduce risk of death at day 32 for V→S flies and day 25 for T→S flies. This predicted inflection time point occurs relatively early in adulthood (before the 50% survival time point) and flies that live beyond this time point have reduced risk when mistranslating compared with controls. We found that the interaction of mistranslation (time-independent covariate) and mating significantly decreased risk in female flies mistranslating from V→S by 1.81-fold compared with what would be expected based on the effects of mating and mistranslation individually (HR = 0.551, *P* = 0.0412; Supplementary Table 4), and a significant 2.24-fold decrease in risk for females mistranslating T→S (HR = 0.445, *P* < 0.0023; Supplementary Table 5). This interaction effect indicates that the risk of death from mating and mistranslating is lessened when the 2 are combined, supporting the notion that mistranslation acts as a synergistic protective buffer against the negative effects of mating on longevity, and particularly so in longer-lived flies.

Lastly, it is possible that the increased longevity of mistranslating mated females is caused by these females not storing male ejaculate or having a reduced investment in reproduction due to energetic allocation toward resolving mistranslation over investing in reproduction (Priest et al. 2008; reviewed in Kressler et al. 2010; reviewed in Liu and Chern 2021). To assess whether mistranslating females are engaging fully in reproduction, we scored egg laying and fertilization to determine if mistranslation affects reproductive output. All mistranslating females and their controls produced eggs (oocytes) (Supplementary Table 3). *Drosophila melanogaster* females store sperm in the seminal receptacle for usage up to 4 days postmating and the spermathecae for usage between 5 d to 3 wk following mating (Dhillon et al. 2020, reviewed in Neubaum and Wolfner 1999). To determine if females produced the same amount of fertilized eggs from both sperm storage organs, we used a linear mixed effects (LME) model on egg laying pattern of V→S females, T→S females, and their respective controls once mated to a wildtype (*CS*) male (Supplementary Table 3). Notably, there was a significant interaction effect between genotype and sperm storage period for V→S mistranslating females and their controls (*P* = 0.0374; Table 3), which was not seen for T→S females. This interaction effect is due to the nonsignificant (via Wilcoxon test) larger and smaller amount of eggs produced from the short-term and long-term sperm storage organs of V→S controls, respectively (Fig. 5a and Table 4). When short-term and long-term egg laying is pooled, there is no significant difference between V→S mistranslating females and their controls, but T→S mistranslating females lay significantly more eggs per day than controls (average 11.0 vs 6.9, respectively; Wilcoxon test, *P* = 0.018; Table 4 and Fig. 5: note that Fig. 5 displays the median and not the mean). This is due to 48.5% more short-term eggs laid by T→S mistranslating compared with control females (12.5 vs 6.4; LME: *P* = 0.0334 Table 3; Wilcoxon test: *P* = 0.018, Table 4; Fig. 5b).

**Fig. 5.**
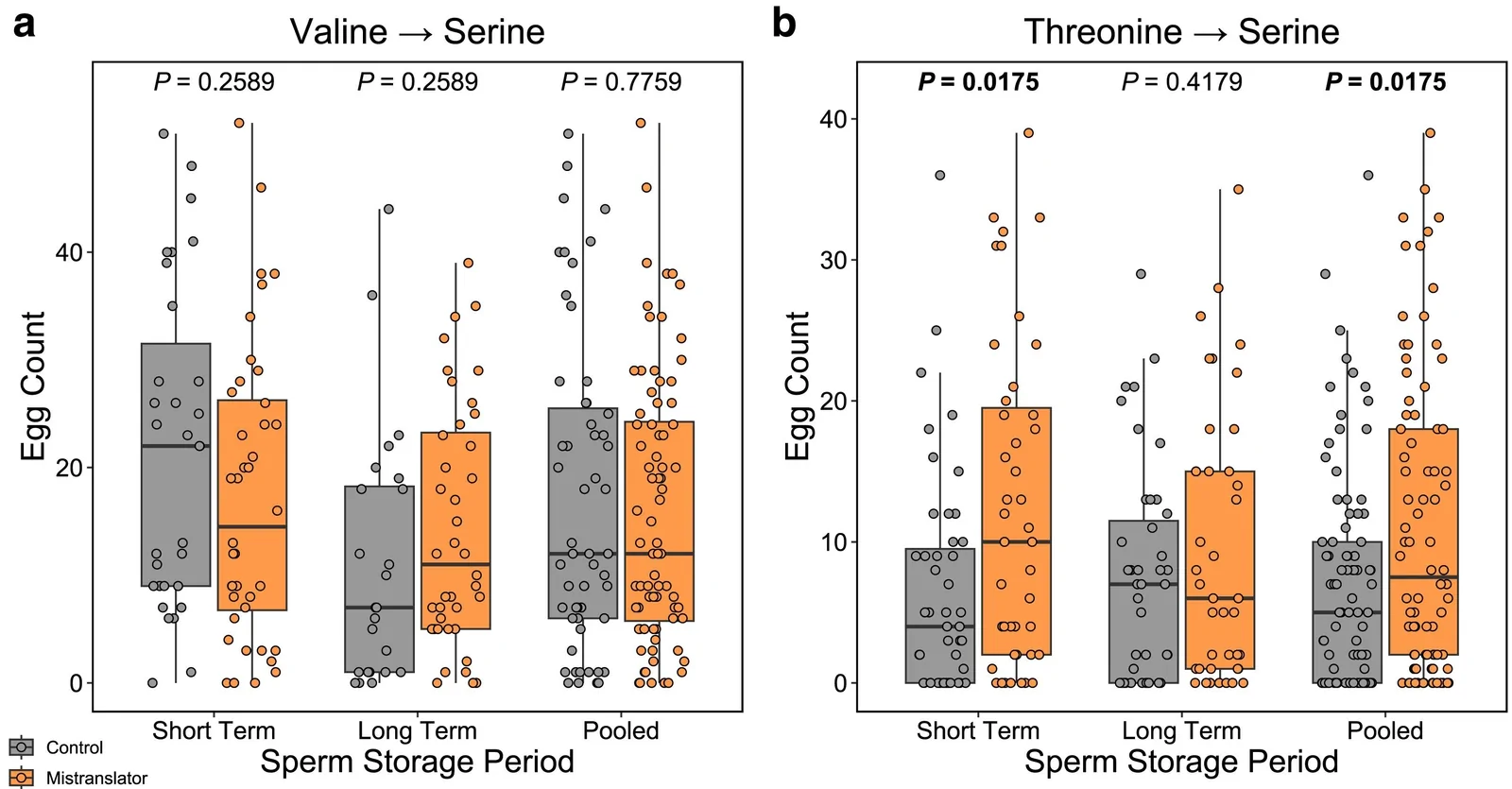
The number of eggs laid each day using sperm from short-term and long-term sperm storage organs, and from the sperm storage organs pooled, for females with an (a) V→S and (b) T→S mistranslating tRNA (orange) compared with controls (gray) after mating with wildtype males. The boxes display the medians and interquartile ranges in the data distribution; circles represent individual samples. Significant *P*-values are shown in bold. Genotypes were compared using Wilcoxon rank-sum tests corrected using BH FDR.

**Table 2.** Cox proportional hazard estimates and corrected *P*-values for all covariates in female V→S and T→S models.

Model	Covariate^a^	Hazard ratio	*P*-value	Adjusted *P*-value^b^
V→S females	Mating status:mistranslation	0.55060	0.01374	**0**.**04122**
	Mating status	4.06061	1.02E-14	**3.05E-14**
	Mistranslation (time-independent)	4.61318	0.00348	**0.00348**
	Mistranslation (time-dependent)	0.56557	7.40E-06	**1.11E-05**
	Initial vial population size	1.00938	0.78688	0.78688
T→S females	Mating status:mistranslation	0.44527	0.00076	**0.00227**
	Mating status	4.80527	1.22E-18	**3.66E-18**
	Mistranslation (time-independent)	10.66775	9.00E-06	**2.70E-05**
	Mistranslation (time-dependent)	0.55873	3.81E-06	**5.72E-06**
	Initial vial population size	1.16577	0.00889	**0.00889**

^a^Reference levels: unmated (mating status), nonmistranslating control (mistranslation).

^b^Significant values are bolded.

**Table 3.** LMEs of egg laying and corrected *P*-values in V→S and T→S mistranslating and control females that were mated to wildtype males.

Model	Covariate^a^	Covariate^b^	*P*-value^c^
V→S females	–	Storage	0.2309
	Storage	–	0.1857
	Storage:mistranslation		**0**.**0374**
T→S females	–	Storage	**0**.**0334**
	Storage	–	0.4147
	Storage:mistranslation		0.1711

^a^Reference levels: long-term sperm storage (storage), nonmistranslating control (mistranslation).

^b^Reference levels: short-term sperm storage (storage), nonmistranslating control (mistranslation).

^c^Significant values are bolded.

**Table 4.** Wilcoxon test of egg laying in the short-term assay, long-term assay, and short-term and long-term assays pooled for V→S and T→S females that were mated to wildtype males.

				*P*-value	
Model	Group 1	Group 2	Assay	Raw	Adjusted^a^
V→S females	Mistranslating	Control	Short	0.17263	0.25895
			Long	0.16684	0.25895
			Pooled	0.77594	0.77594
T→S females	Mistranslating	Control	Short	0.00778	**0**.**01752**
			Long	0.41792	0.41792
			Pooled	0.01168	**0**.**01752**

^a^ *P*-value corrected for multiple testing using BH FDR. Significant values are bolded.

To confirm that eggs were fertilized, we tested for fertilization status halfway through short-term sperm storage (day 2 postmating) and long-term sperm storage (day 8 postmating) assays and found that 100% of eggs that were produced were fertilized. Results indicate that the increased longevity observed for mistranslating females is not due to a failure of sperm storage or lack of investment in reproduction, at least via the measures scored here.

## Discussion

According to the Disposable Soma Theory, reproduction leads to a reduction in lifespan due to the physiological cost of gamete production and favoring of gametic tissue proteostasis over somatic tissues (Evans et al. 1997; Kirkwood 1977; Kirkwood and Holliday 1997; reviewed in Flatt 2011). The cost of reproduction would be more pronounced in females than in males due to the higher energetic cost of female egg production than male sperm production. Based on the Disposable Soma Theory, we predicted that a disruption to proteostasis due to mistranslation would further compound the detrimental effects of mating on lifespan. When we assessed the impact of reproduction and mistranslation on *D. melanogaster* lifespan, we found that, counter to expectation, mistranslation caused by 2 different tRNA^Ser^ variants both increased mated female lifespan. There were no differences in egg laying between V→S mistranslating females and their controls, and T→S mistranslating females actually lay more eggs than their respective controls, further indicating that the increase in longevity observed cannot be attributed to a general failure or reduction in reproduction due to mistranslation. However, it is possible that other measures of reproductive investment, such as egg size, may reveal differences between mistranslating females and controls and warrant further examination. Mistranslation increased mated female lifespan to be equal to that of mated males, thus mistranslation appears to protect females from the detrimental impact of reproduction. Notably, mistranslation causes a synergistic and significant decrease in risk of death of female flies that are mistranslating and mated. Mistranslation therefore benefits female lifespan even when compounded with other stressors, indicating its potential as an evolutionary mechanism in multicellular organisms to improve adult fitness (Bacher et al. 2007; Netzer et al. 2009; Vargas-Rodriguez et al. 2021).

One limiting factor of our study is that we did not control for mating frequency during the longevity assay, which could impact longevity if different genotypes have different mating frequencies. For example, a higher mating frequency in early life has been shown to increase mortality in *D. melanogaster* females (Barnes et al. 2008; Travers et al. 2015). While male mortality was not different between the genotypes, and thus the number of males available to mate were similar over time, future studies that control for how often mating occurs throughout lifespan could determine if this is a contributing factor toward the longevity differences we observed. We also note that although we see significant effects due to mistranslation, we did not find the same increased longevity for T→S mistranslating females previously reported (Isaacson et al. 2024b) for these flies. It may be that the significant effect of initial vial population size (Table 2) confounded the longevity results. It is also possible that background genetic effects influencing longevity have arisen in this strain in the time between that earlier study and the one reported here, that the smaller sample size in the current study was insufficient to detect the T→S longevity increase, or that the effect on longevity is sensitive to subtle environmental differences that could be present between earlier assays and the ones here. Since T→S mistranslated at approximately twice the amount as V→S (1.0% vs 0.5% per codon, respectively; Isaacson et al. 2024b), then suppressors of mistranslation are more likely to have arisen in the T→S strain (Hoffman et al. 2017). If mistranslation is being partially suppressed in T→S flies to where it is removing the increased lifespan effect in unmated females that would mean that our findings are likely under-representing the potential improved lifespan effects for mated females.

The removal of the impact of reproduction on lifespan in the presence of mistranslation is surprising. What could explain mated female lifespan being longer with mistranslation than without? One mechanism could be heightened stress-response pathways in the presence of mistranslation, causing the females to be “primed” to respond to cellular stressors that arise with reproduction. A previous evaluation of gene expression with mistranslation in *Drosophila* did not find upregulation of stress-response genes (Isaacson et al. 2024a), but stress-response pathways are often increased at the protein level rather than transcript level (eg Kaarniranta et al. 1998; Harding et al. 2000; reviewed in Advani and Ivanov 2019). Stress-response pathway levels may also be impacted by fragmentation of tRNAs, which can occur in response to stress, but whose effect would not be detected via sequencing of mRNAs. Fragmentation due to stressors depletes the amount of a tRNA available for translation (Huh et al. 2021), altering the tRNA pool in a way that causes increased levels of required proteins for different stress responses (Torrent et al. 2018). Thus, proteomic analysis may identify some of the stress-response pathways that differ between mated and unmated mistranslating flies.

Flies also have low levels of naturally occurring mistranslation (Isaacson et al. 2024b). When we induce substantial levels of mistranslation, this creates a strong selective pressure on the flies. Since mistranslation can lead to high rates of developmental mortality and physical abnormalities (Isaacson et al. 2024b), flies with either the ability to suppress mistranslating tRNAs or to increase proofreading of protein production would have a higher chance of surviving to successfully reproduce. It may be that adults with a reduction of overall mistranslation, which could affect both transgenically induced and naturally occurring mistranslating tRNA variants, leads to increased longevity with particularly strong benefits during the protein-intensive process of reproduction. In other words, it may be that the benefit of mistranslation caused by our transgenes is that it selects for flies that have suppressors of mistranslation, including naturally occurring mistranslation. Whether this is the case could be explored in the future through assessing the amount of mistranslation present in the longest-lived mistranslating vs wildtype flies.

From a selection standpoint, positive selection for increased longevity due to the presence of a mistranslating tRNA would conflict with the negative effects of mistranslation during development. Further studies exploring how mistranslating flies respond to other stressors, or how mistranslation might influence physiology and metabolism, could help elucidate the potentially complex interplay between selection pressures and mistranslation. Additionally, there may be tissue-specific effects of mistranslation on lifespan that are not captured by the ubiquitous expression model used here. Therefore, further studies with mistranslation occurring only after completion of development, and assessment of the impacts of mistranslation confined to particular tissues, will help elucidate the underlying basis of the positive effect of mistranslation on lifespan.

## Supplementary Material

jkag216_Supplementary_Data

## Data Availability

Fly lines and plasmids are available upon request. The authors affirm that all data necessary for confirming the conclusions of the article are present within the article, figures, and Supplementary material. Supplementary Fig. 1 and Tables 1–5 are appended to the manuscript. The Supplementary Table 6 file contains all raw data. Supplementary File 1 contains the R code used to analyze all raw data. Supplemental material available at G3 online.
