# Supplementary material for: Mistranslation protects against lifespan reduction due to mating in Drosophila melanogaster females
Source: G3 (Bethesda). 2026 Aug 12;16(10):jkag216. doi: 10.1093/g3journal/jkag216 (PMC13643807; doi:10.1093/g3journal/jkag216)
Supplement: Supplementary file 1 [file jkag216_supplementary_data.zip › Supplemental_Figures_and_Tables_G3-2026-407108.pdf]

SUPPLEMENTARY FIGURES AND TABLES

Supplementary Figure 1. Longevity assay setup on a given day. Each male is depicted in the vials by the "♂" symbol and females are depicted by the "♀" symbol. The number of each fly in each vial is displayed beneath the vial. For a given set, vials contained a total of 4, 6, or 8 flies, with all vials within that set having the same number. These examples display a total of a) 4 flies and b) 8 flies.

Suppl Fig 1 Alt Text: Vials of the longevity assay setup. Panel a displayed the setup using 4 flies per vial. Panel 8 displays the setup using 8 flies per vial. There are 8 vials: 4 mistranslating and 4 control. Each 4 vials are resented by males, females, mated vial 1, and mated vial 2.

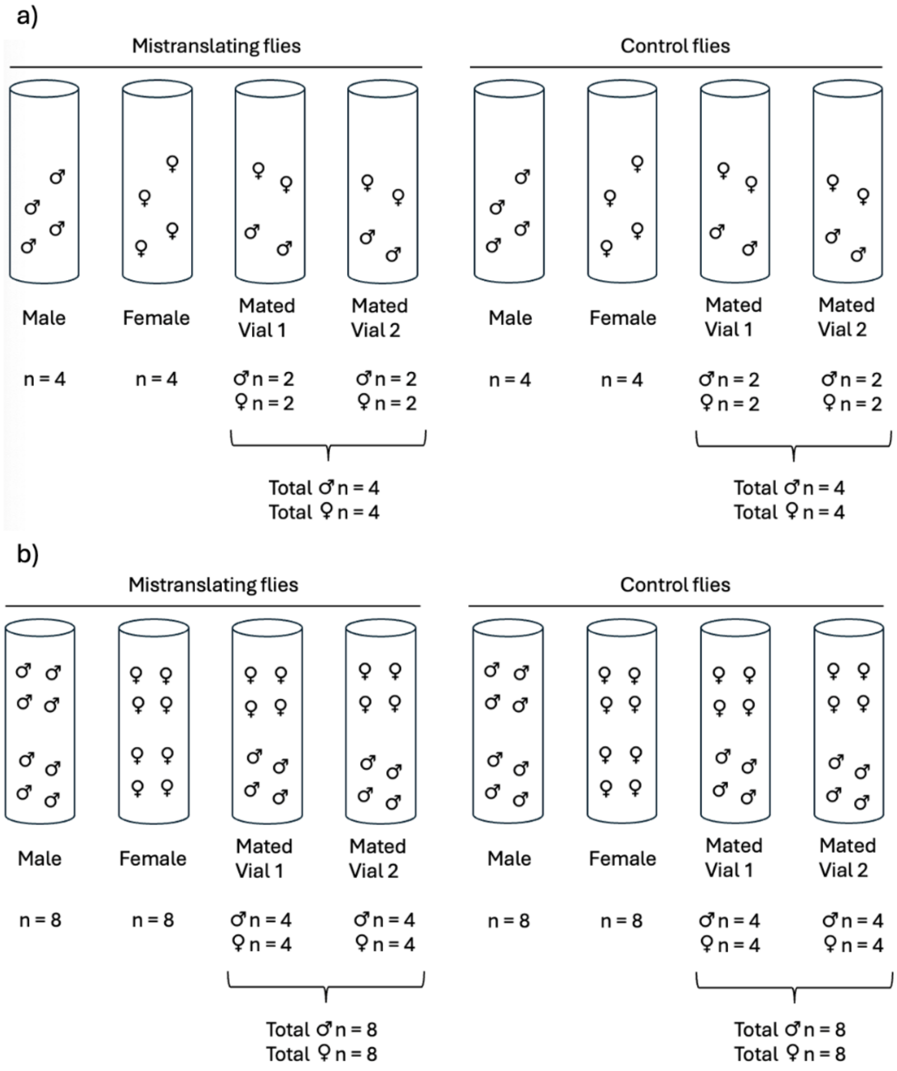

Supplementary Table 1. Number of vials and flies in the longevity assay. The total number (n) of flies before censoring is shown, along with the number of data points that were censored.

Model	Sex	Mating Status	Genotype	Vials	Flies	Censored Flies
V→S	F	Unmated	Mistranslator	18	80	2
			Control	18	82	0
		Mated	Mistranslator	36	82	0
			Control	36	82	0
	M	Unmated	Mistranslator	18	81	1
			Control	18	81	1
		Mated	Mistranslator	36	82	0
			Control	36	81	1
T→S	F	Unmated	Mistranslator	16	76	4
			Control	16	79	1
		Mated	Mistranslator	32	79	1
			Control	32	80	0
	M	Unmated	Mistranslator	16	80	0
			Control	16	80	0
		Mated	Mistranslator	32	80	0
			Control	32	80	0

Supplementary Table 2. *P*-values of comparison groups. The table displays the unadjusted and BH-adjusted *P*-values for each mistranslating line (V→S and T→S) and their controls, categorised by sex (Males and Females) and mating status (mated and unmated). Each reference's *P*-value is separated into their comparisons: Male x Female, MT x Control, and Mated x Unmated. Adjusted *P*-values that are bolded remained significant after adjustment.

**Model**	**Comparison factor**	**Constant Factors**			***P-value***	
		Sex	*Mistranslation*	*Mating status*	Raw	Adjusted
V→S	*Sex*	–	control	unmated	0.92010	0.92010
		–	mistranslating	unmated	0.00790	**0.01724**
		–	control	mated	<0.0001	**<0.0001**
		–	mistranslating	mated	0.41770	0.45567
	Mistranslation	M	–	unmated	0.17010	0.24014
		F	–	unmated	<0.0001	**<0.0001**
		M	–	mated	0.26900	0.33979
		F	–	mated	<0.0001	**<0.0001**
	Mating status	M	control	–	0.28350	0.34020
		M	mistranslating	–	0.16840	0.24014
		F	control	–	<0.0001	**<0.0001**
		F	mistranslating	–	<0.0001	**<0.0001**
T→S	*Sex*	–	control	unmated	0.00030	**0.00090**
		–	mistranslating	unmated	0.01410	**0.02820**
		–	control	mated	0.00490	**0.01176**
		–	mistranslating	mated	0.34410	0.39326
	Mistranslation	M	–	unmated	0.08530	0.14623
		F	–	unmated	0.59010	0.61576
		M	–	mated	0.10340	0.16544
		F	–	mated	0.00040	**0.00107**
	Mating status	M	control	–	0.07810	0.14418
		M	mistranslating	–	0.19920	0.26560
		F	control	–	<0.0001	**<0.0001**
		F	mistranslating	–	<0.0001	**<0.0001**

Supplementary Table 3. Number of eggs laid by each female for each cross after a single mating to a wildtype male. Days 1–4 represent short-term egg laying and days 7–10 represent long-term egg laying. NA represents flies that were harmed or lost during transfer.

		**Number of Eggs Laid**							
Female	#	Day 1	Day 2	Day 3	Day 4	Day 7	Day 8	Day 9	Day 10
V→S	1	24	4	12	3	12	5	9	6
	2	27	20	16	34	0	29	17	22
	3	30	8	9	9	7	10	18	5
	4	20	46	9	3	29	13	39	8
	5	0	8	6	7	2	15	1	7
	6	1	9	28	26	0	12	35	0
	7	0	29	38	13	1	7	32	28
	8	0	3	37	19	5	8	25	26
	9	52	12	21	19	19	24	23	8
	10	24	2	38	23	5	20	34	5
V→S Control	1	40	13	23	28	23	10	19	5
	2	45	7	7	9	NA	NA	NA	NA
	3	39	0	1	NA	NA	NA	NA	NA
	4	51	28	12	40	1	18	7	18
	5	35	12	9	9	3	1	44	12
	6	41	22	6	26	1	36	11	6
	7	22	25	6	9	0	0	0	7
	8	48	11	24	26	1	20	22	1
T→S	1	1	26	10	24	0	15	18	NA
	2	31	20	4	13	0	2	6	2
	3	6	32	10	NA	NA	NA	NA	NA
	4	0	0	0	0	1	0	6	13
	5	0	0	0	2	0	0	7	5
	6	19	33	8	2	2	35	15	1
	7	18	16	4	2	5	18	15	8
	8	12	39	17	7	0	18	23	5
	9	2	33	19	4	1	23	24	1
	10	24	21	15	4	1	26	22	2
	11	11	31	13	4	0	14	9	10
T→S Control	1	0	12	5	10	0	0	0	0
	2	19	9	5	2	7	13	8	8
	3	1	9	0	0	0	9	0	21
	4	4	8	3	0	0	2	17	8
	5	4	0	0	5	2	7	13	0
	6	16	12	9	7	0	0	6	12
	7	2	0	0	0	0	0	7	8
	8	0	0	0	0	0	2	5	NA
	9	12	25	3	3	1	29	8	13
	10	0	22	2	18	2	21	20	10

11	5	9	0	NA	NA	NA	NA	NA
12	0	15	36	10	11	23	8	18

Supplementary Table 4. Cox proportional hazard estimates and *P*-values for all covariates across sex-pooled, male, and female V→S survival models.

V→S	Covariate	Hazard ratio	*P*-value	Adjusted *P*-value†
Interaction terms	mating status:sex:mistranslation	0.43436	**0.00901**	–
	mating status:sex	3.81052	**5.01E-9**	–
	sex:mistranslation	0.66801	**0.07002**	–
	mating status:mistranslation	1.19962	0.41414	0.41414
	– *male*	1.20095	0.41366	0.41414
	– *female*	0.55060	**0.01374**	**0.04122**
Main terms	sex	1.07818	0.63047	–
	mating status	1.16402	0.33393	0.34872
	– *male*	1.15945	0.34872	0.34872
	– *female*	4.06061	**1.02E-14**	**3.05E-14**
	mistranslation (time independent)	8.45394	**9.04E-07**	**2.71E-06**
	– *male*	23.16539	**0.00024**	**0.00037**
	– *female*	4.61318	**0.00348**	**0.00348**
	mistranslation (time dependent)	0.54197	**2.63E-09**	**7.88E-09**
	– *male*	0.42612	**4.38E-05**	**0.00004**
	– *female*	0.56557	**7.40E-06**	**0.00001**
	initial vial population size	1.05226	**0.03882**	0.05823
	– *male*	1.10624	**0.00448**	**0.01345**
	– *female*	1.00938	0.78688	0.78688

† P-value corrected for multiple testing for all one-way and two-way interactions using Benjamini-Hochberg false discovery rate. Significant values are bolded.

Reference levels: unmated (mating status), male (sex), non-mistranslating control (mistranslation)

Supplementary Table 5. Cox proportional hazard estimates and *P*-values for all covariates across sex-pooled, male, and female T→S survival models.

T→S	Covariate	Hazard ratio	*P*-value	Adjusted *P*-value†
Interaction terms	mating status:sex:mistranslation	0.58387	0.09459	–
	mating status:sex	3.09235	**8.10E-07**	–
	sex:mistranslation	1.47627	0.08673	–
	mating status:mistranslation	0.82055	0.37985	0.47595
	– *male*	0.85158	0.47595	0.47595
	– *female*	0.44527	**0.00076**	**0.00227**
Main terms	sex	0.51179	**0.00003**	–
	mating status	1.41349	**0.02888**	**0.03343**
	– *male*	1.40130	**0.03343**	**0.03343**
	– *female*	4.80527	**1.22E-18**	**3.66E-18**
	mistranslation (time independent)	4.51845	**0.00003**	**0.00005**
	– *male*	2.74026	**0.03315**	**0.03315**
	– *female*	10.66775	**0.00001**	**0.00003**
	mistranslation (time dependent)	0.62727	**6.56E-08**	**1.97E-07**
	– *male*	0.72124	**0.00625**	0.00625
	– *female*	0.55873	**3.81E-06**	**0.00001**
	initial vial population size	1.19454	**0.00002**	**0.00005**
	– *male*	1.21652	**0.00071**	**0.00106**
	– *female*	1.16577	**0.00889**	**0.00889**

† P-value corrected for multiple testing for all one-way and two-way interactions using Benjamini-Hochberg false discovery rate. Significant values are bolded.

Reference levels: unmated (mating status), male (sex), non-mistranslating control (mistranslation)
